# 92-Gene Molecular Profiling in Identification of Cancer Origin: A Retrospective Study in Chinese Population and Performance within Different Subgroups

**DOI:** 10.1371/journal.pone.0039320

**Published:** 2012-06-22

**Authors:** Fei Wu, Dan Huang, Lisha Wang, Qinghua Xu, Fang Liu, Xun Ye, Xia Meng, Xiang Du

**Affiliations:** 1 Department of Pathology, Fudan University Shanghai Cancer Center, Shanghai, People’s Republic of China; 2 Department of Oncology, Shanghai Medical College, Fudan University, Shanghai, People’s Republic of China; 3 Institut Mérieux Laboratory, Fudan University Shanghai Cancer Center, Fudan University Shanghai Cancer Center, Shanghai, People’s Republic of China; 4 Institutes of Biomedical Sciences, Fudan University, Shanghai, People's Republic of China; National Cancer Center, Japan

## Abstract

**Background:**

After cancer diagnosis, therapy for the patient is largely dependent on the tumor origin, especially when a metastatic tumor is being treated. However, cases such as untypical metastasis, poorly differentiated tumors or even a limited number of tumor cells may lead to challenges in identifying the origin. Moreover, approximately 3% to 5% of total solid tumor patients will not have to have their tumor origin identified in their lifetime. The THEROS CancerTYPE ID® is designed for identifying the tumor origin with an objective, rapid and standardized procedure.

**Methodology and Principal Findings:**

This is a blinded retrospective study to evaluate performance of the THEROS CancerTYPE ID® in a Chinese population. In total, 184 formalin-fixed paraffin-embedded (FFPE) samples of 23 tumor origins were collected from the tissue bank of Fudan University Shanghai Cancer Center (FDUSCC). A standard tumor cell enrichment process was used, and the prediction results were compared with reference diagnosis, which was confirmed by two experienced pathologists at FDUSCC. All of the 184 samples were successfully analyzed, and no tumor specimens were excluded because of sample quality issues. In total, 151 samples were correctly predicted. The agreement rate was 82.1%. A Pearson Chi-square test shows that there is no difference between this study and the previous evaluation test performed by bioTheranostics Inc. No statistically significant decrease was observed in either the metastasis group or tumors with high grades.

**Conclusions:**

A comparable result with previous work was obtained. Specifically, specimens with a high probability score (>0.85) have a high chance (agreement rate = 95%) of being correctly predicted. No performance difference was observed between primary and metastatic specimens, and no difference was observed among three tumor grades. The use of laser capture micro-dissection (LCM) makes the THEROS CancerTYPE ID® accessible to almost all of the cancer patients with different tumor statuses.

## Introduction

Approximately 10% to 15% of cancer patients are defined as metastatic cancer patients when they are first diagnosed [1]. Information on the tumor origin is valuable in treatment decisions. The accurate diagnosis of the primary site allows clinicians to determine the stage of cancer; surgery could be applied in some cases, and further radio- and chemotherapy or site-specific therapy could also be of benefit for the patients. A retrospective study with 879 patients showed an increase in survival time in patients for whom the primary site was identified [2]. However, untypical metastasis or poorly differentiated tumors can present challenges in identifying the tumor origin.

Increased efforts have been made to trace the origin of a tumor using different skills and technologies. In some cases, an experienced pathologist will know the answer by examining hematoxylin and eosin-stained (H&E) slides. Immunohistochemistry (IHC) is a routine test in the pathology department and will sometimes provide helpful information at the protein level.

However, even with an expanding panel of antibodies, it is still not possible to obtain a convincing conclusion with IHC independently, and this diagnosis could be more difficult in high-grade tumors. A meta-analysis of four studies showed that IHC correctly identified the tumor origin of 66% of specimens [3]. Some DNA tests, including loss of heterozygosity (LOH) analysis, microsatellite analysis and oncogene mutation analysis, could be used to show the clonal origin of a malignancy. With the evolution of diagnostic criteria, tools and technology, most of the primary cancer lesions could finally be identified after a series of time-consuming procedures. However, approximately 3% to 5% of total solid tumor patients will not be able to have their tumor origin accurately diagnosed before treatment starts or even in their lifetime [4], [5].

Recently, different molecular analyses for the expression profiling of mRNA or miRNA were developed to identify the primary site [6], [7], [8], [9] using microarrays or real-time PCR technology. Several critical mRNAs or miRNAs were profiled in the metastatic site of tumors, and the results indicated the possible primary site. Validation results for these molecular assays were published, and the success rates were approximately 75.6% to 89%.

The THEROS CancerTYPE ID® is a real-time PCR-based test that can be used to identify the origin of metastatic cancer and determine the pathological type of a solid tumor. A total of 92 genes, including 5 reference and 87 tumor-specific genes, were detected in FFPE samples from slides.

The 1^st^ version of THEROS CancerTYPE ID® was designed and tested in 2006 [6]. A total of 578 tumor samples were selected to develop a comprehensive database. A possibility score was given after the test as a measurement of how similar the sample tested was to 32 tumor origins and histological subtypes in the reference database. Validation results showed an 87% success rate in the classification of 32 tumor types and within 119 FFPE samples. Further validation was demonstrated in the identification of specimens from 20 actual CUP patients at least 2 months earlier than their latent primary recognition, and most of these specimens were from poorly differentiated tumors. This study showed that 15 of the 20 samples were accurately classified and corresponded to the latent primary sites later identified [10].

THEROS CancerTYPE ID® (Version 2) was subsequently developed. The reference tumor database was expanded to 2,206 samples, and the associated algorithm was modified to enable the prediction of 30 main tumor types and 54 histological subtypes. More importantly, a tumor enrichment method, laser capture micro-dissection (LCM), was used. This addition makes the technology applicable when limited tumor cells are available. In a separate test set of 187 FFPE tumor samples representing 28 of the 30 main cancer types, THEROS CancerTYPE ID® (Version 2) showed an overall sensitivity of 83% [11].

In this study, we aim to evaluate the performance of the THEROS CancerType ID® (Version 2) within Chinese population. We conducted a blinded retrospective study in which 184 FFPE samples from FDUSCC were selected, real-time PCR was performed in Shanghai, and the generated raw data were analyzed by bioTheranostics in San Diego. The prediction results were generated by bioTheranostics. The assay performance within several subgroups, including different technologies and tumor characteristics, was compared.

## Materials and Methods

### Study Design

This is a blinded retrospective study to evaluate the performance of the THEROS CancerTYPE ID® in Chinese population. FFPE samples of 23 tumor origins were selected from FDUSCC. Clinical history and H&E slides were confirmed by at least 2 experienced pathologists. Tumor cells were dissected from FFPE slides by scraping or LCM. After overnight digestion with proteinase K, a standard protocol was applied to isolate and reverse transcribe RNA. A 92-gene Taqman real-time PCR panel was then used for each sample. PCR data were generated and sent to the CLIA lab at bioTheranostics Inc. for analysis. The data were generated using a previously described method without knowing any of the clinical information, except for gender and organ location in which the tissues were obtained [6], [11].

A total of 184 samples of 23 tumor main types were selected for this study, and these samples are described in Table 1. These samples were separated into 2 groups for analysis according to the difficulty of clinical assessment and practice: 139 samples of 17 tumor types were classified into groups in which the primary site (n = 102, 73.4%) and metastasis site (n = 37, 26.6%) were identified. The remaining 45 samples of 6 tumor types, including sarcoma, neuroendocrine, mesothelioma, skin, melanoma and lymphoma cancer, could arise in many parts of the body or in multiple organs; therefore, it is not easy to accurately determine the primary site or origin of the tumor. For example, lymphoma could be found at both sides of the diaphragm when diagnosed, and the exact origin could not be determined. In addition, these 6 types of tumors are not in the report of the THEROS CancerTYPE ID®. We decided to compare these 45 samples separately for their primary and metastatic tumors. The patient clinical characteristics are illustrated in Table 2.

**Table 1:** No. of cases for each cancer type and primary or metastasis site.

Cancer Type	No. ofCases	Primary Site	Metastasis Site
**Ovary**	14	10	4
Mucinous adenocarcinoma	4		
Clear cell adenocarcinoma	1		
Endometrioid	1		
Serous	8		
**Sarcoma**	**13**	**/**	**/**
**Gastro esophageal**	**12**	**7**	**5**
Adenocarcinoma	11		
Squamous	1		
**Head & Neck**	**10**	**7**	**3**
Salivary	6		
squamous	4		
**Urinary Bladder**	**10**	**6**	**4**
TCC	9		
Adenocarcinoma	1		
**Germ-cell**	**10**	**10**	**0**
Germinomatous	2		
Non-Germinomatous	4		
Mixed	4		
**Breast**	**10**	**7**	**3**
Ductal carcinoma	6		
Lobular carcinoma	2		
Mucinous adenocarcinoma	2		
**Thyroid**	**10**	**6**	**4**
Papillary	4		
Follicular	2		
Medullary	4		
**Neuroendocrine**	**9**	**/**	**/**
Gastrointestinal carcinoid	4		
Lung carcinoid	1		
Lung large cell	1		
Lung small cell	2		
Pancreatic islet cell carcinoid	1		
**Kidney**	**8**	**8**	**0**
Chromophobe	2		
Clear cell	5		
Transitional cell	1		
**Lung**	**8**	**7**	**1**
Large cell	4		
Squamous	4		
**Intestine**	**8**	**2**	**6**
Colon adenocarcinoma	8		
**Mesothelioma**	**7**	**/**	**/**
**Pancreas**	**7**	**6**	**1**
Adenocarcinoma	7		
**Prostate**	**7**	**7**	**0**
Adenocarcinoma	7		
**Endometrium**	**6**	**3**	**3**
Adenocarcinoma	2		
Adeno-squamous carcinoma	1		
Clear cell carcinoma	2		
**Skin**	**6**	**/**	**/**
Basal cell carcinoma	3		
Squamous	3		
**GIST**	**6**	**6**	**0**
**Liver**	**6**	**5**	**1**
**Gallbladder**	**5**	**4**	**1**
Adenocarcinoma	5		
**Melanoma**	**5**	**/**	**/**
**Lymphoma**	**5**	**/**	**/**
MALT	2		
DLBCL	2		
Burkitt	1		
**Adrenal**	**2**	**1**	**1**
Pheochromocytoma	1		
Cortical carcinoma	1		
**Total**	**184**	**102**	**37**

**Table 2:** Patient clinical characters.

Patient Gender		
Male	90	
Female	94	
**Tumor Grade**		
I (Low grade)	12	
II (Intermediate grade)	28	
III (High grade)	50	
Not specified	94	
**Primary and metastasis**		
Primary	102	
Metastasis	37	
Not specified	45	

### Patients and Tumor Specimens

A total of 184 tumor samples were obtained from the tissue bank of FDUSCC. Tumor types that were not in the THEROS CancerType ID® diagnostic list were not included, and FFPE blocks before 2008 were also not included in this study. Diagnoses were made based on necessary medical history review, physical evaluation, imaging procedures and full pathologic workup, including H&E staining. All of the cases were reviewed and diagnosed by at least two pathologists.

For each sample, a minimum of 300 tumor cells should be obtained after dissection. No other special inclusion criteria, such as weight, tumor representation and minimal necrosis rate, were required due to the dissection technology used.

### FFPE Slides and Tumor Cells Enrichment

Samples were embedded in paraffin with a standard FFPE protocol and stored in the tissue bank of FDUSCC. The H&E slides for each tumor block were examined to confirm the existence of tumor cells. Samples with a large tumor area and high tumor cells content without necrosis are prepared for manual dissection. Other samples with large interfering areas, such as normal cells, necrotic areas, fibrocytes, and lymphocyte infiltrations, or with low tumor representation and multiple discrete tumor cells under the microscope were labeled for LCM [12]. FFPE blocks were prepared for each treatment as three unstained 10-µm glass slides (for scrape) or membrane slides (for LCM) and one H&E-stained slide. Further treatment included deparaffinization, circling the area of the tumor (only for scrape) or staining (only for LCM) and proteinase K digestion overnight.

In total, the tumor cells of 127 specimens were dissected by LCM, and the remaining 57 specimens were scraped.

### RNA Extraction and Pre-amplification

After proteinase K (Life Technologies, Inc.) treatment overnight (16–20 h), RNA extraction was performed with a Zymo RNA Extraction Kit (ZYMO research) according to the recommended protocol. Then, 10 µl of purified RNA was treated by DNAse (Life Technologies, Inc.) to eliminate genome DNA contamination in the PCR process. After reverse transcription by poly-T and random hexamer primers, a pre-amplification step was performed with an ABI pre-AMP kit (Life Technologies, Inc.).

### TaqMan PCR Assay

THEROS CancerTYPE ID® (Version 2) is a Taqman-based real-time PCR assay that detects the expression level of 92 genes and distinguishes 30 tumor types. Within these 92 genes, 5 reference genes with stable expression across the broad spectrum of tissues are used for scaling and QC. The remaining 87 genes were expressed in multiple tumors. Approximately 80% of these genes have functional annotation, including DNA-binding transcription factors, cell membrane proteins and several well-characterized tumor markers [6].

The assay was processed with prefabricated ABI 384 plates. Four samples were applied to each 384 plate. To control the quality of experiments, a negative control and a positive control are used with each set of experiments. The negative control replaces the real sample with H_2_O, and the positive control is a universal human RNA purchased from Stratagene, Inc. Primer pairs and MGB Taqman probes were designed to produce an amplicon of less than 80 bps and synthesized by Sangon Biotech (Shanghai). The aliquoting of pre-amplified samples was performed in a 10 µl volume in a prefabricated 384 plate. Amplifications were performed with an ABI 7900HT RT-PCR system with the following conditions: 50°C for 2 minutes, 95°C for 10 minutes, and 45 cycles of 95°C for 15 seconds and 60°C for 1 minute. The raw data were generated with default settings.

### Data Analysis

Raw data were identified with biopsy site and gender and sent to bioTheranostics, Inc. without any other clinical information. With the KNN (K nearest neighbor) algorithm, the expression profiles of the samples were compared with a pre-established database. A standard report was generated for each sample and sent back. The report included a prediction result with a probability score indicating the similarity of the tested samples to the profiling data in the database. The standard reports and clinical diagnosis were compared to calculate the agreement rate. Statistics between several subgroups, such as high and low RNA quality, LCM and scraping, primary site and metastasis site, well and poor differentiation, were also described to demonstrate the performance of the THERO CancerType ID®. The performance of THERO CancerType ID® Version 1 and Version 2 was also compared and described.

## Results

### Assay Performance

The 184 samples were composed of 23 cancer types within the THEROS CancerTYPE ID® working list. We calculated the rate of agreement between the reference diagnosis and THEROS CancerTYPE ID® prediction results (Table 3). An agreement rate of 82.1% (151/184) was achieved for all of the samples. Moreover, the specimens from adrenal, breast, germ cell, GIST, intestinal, liver, lymphoma, prostate and thyroid cancer were 100% correctly classified. The specimens from neuroendocrine, kidney, lung, skin, gallbladder, melanoma and urinary bladder cancer showed an agreement rate higher than 80%. For each type of cancer, we calculated the sensitivity, specificity, positive predictive value (PPV) and negative predictive value (NPV) with the following formulas:

**Table 3:** CancerType ID^®^ performance on 184 Chinese tumor specimens.

Cancer Type	No. of cases	Sensitivity	Specificity	PPV	NPV
**Adrenal**	2	1.000	0.995	0.667	1.000
**Breast**	10	1.000	0.994	0.909	1.000
**GIST**	6	1.000	1.000	1.000	1.000
**Intestine**	8	1.000	0.994	0.889	1.000
**Liver**	6	1.000	1.000	1.000	1.000
**Lymphoma**	5	1.000	0.994	0.833	1.000
**Prostate**	7	1.000	1.000	1.000	1.000
**Thyroid**	10	1.000	1.000	1.000	1.000
**Germ-cell**	10	1.000	1.000	1.000	1.000
**Neuroendocrine**	9	0.889	0.983	0.727	0.994
**Kidney**	8	0.875	0.989	0.778	0.994
**Lung**	8	0.875	0.983	0.700	0.994
**Skin**	6	0.833	0.994	0.833	0.994
**Gallbladder**	5	0.800	0.983	0.571	0.994
**Melanoma**	5	0.800	0.994	0.800	0.994
**Urinary Bladder**	10	0.800	0.971	0.615	0.988
**Sarcoma**	13	0.769	1.000	1.000	0.983
**Pancreas**	7	0.714	0.994	0.833	0.989
**Head & Neck**	10	0.700	1.000	1.000	0.983
**Ovary**	14	0.643	0.976	0.692	0.971
**Gastro esophageal**	12	0.583	0.988	0.778	0.971
**Mesothelioma**	7	0.571	1.000	1.000	0.983
**Endometrium**	6	0.333	0.994	0.667	0.978

Sensitivity: the ability to predict true positives  =  true positives/total observed positives.

Specificity: the ability to predict true negatives  =  true negatives/total observed negatives.

PPV: fraction of true positives among the predictive positives  =  true positives/total number of predicted positives.

NPV: fraction of true negatives among the predictive negatives  =  true negatives/total number of predicted negatives.

### Average Reference Gene Ct (ARG Ct) Value and Probability Score

In this study, RNA was extracted from FFPE slides by either scraping or LCM. These methods usually result in unfavorable RNA quantity and integrity. Because of the scarcity of these samples, we do not check the RNA quality after it was extracted. After real-time PCR is performed, the average Ct value of 5 reference genes is used as an indication of RNA quality. Low RNA quantity or integrity will lead to a higher Ct, indicating a non-optimal condition of the RNA materials.

**Figure 1:**
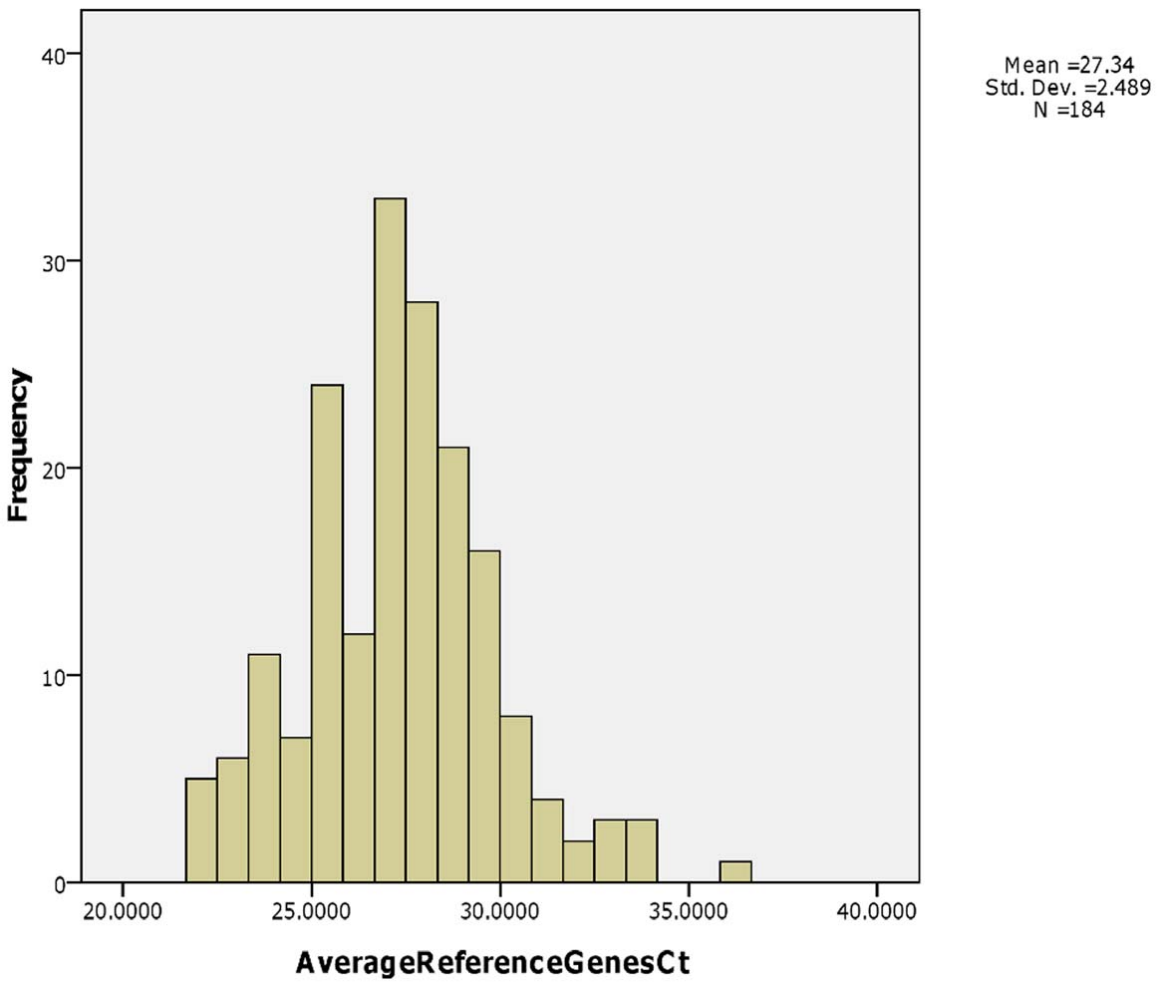
Histogram of average reference genes and number of samples. A histogram of the number of samples versus average reference genes Ct value shows a normal distribution. It is possibly due to the nature of sample quality (both quantity and integrity) from LCM. Also this normal distribution supports viewpoint that ARG Ct value is a good indicator for sample quality.

These 184 tested samples have a wide range of average reference gene Ct values, from 21.7 to 36.3 (Figure 1). The Kolmogorov-Smirnov test showed that these ARG Ct values were normally distributed (*P* = 0.2). The mean and median were 27.34 and 27.36, respectively. A total of 29 samples showed ARG Ct values within the range of 21.7 to 25, and 26 of them were correctly predicted (89.7%). For the range of 25 to 30, 107 of 132 (81.1%) samples were correctly predicted. For the samples having a higher ARG Ct (>30), 18 of 23 samples (78.3%) were correctly predicted. A Pearson Chi-square test showed no significant difference between these 3 groups (*P* = 0.484).

**Figure 2:**
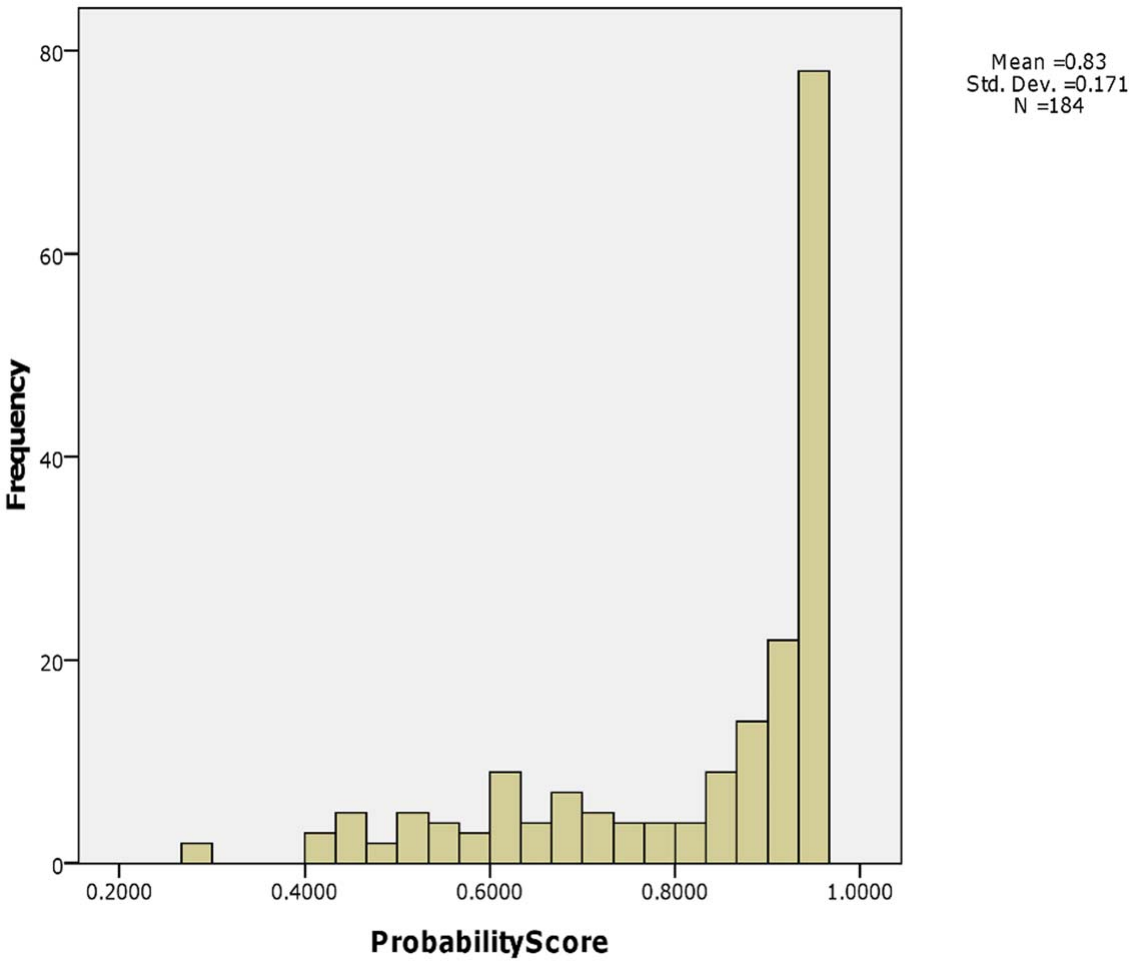
Histogram of probability score and number of samples. A histogram of the number of samples versus probability score shows a highly biased distribution. Most of the samples tested have a probability score higher than 0.85 and got a very high agreement rate (95%).

The probability score is an indicator of classification certainty. In our test, the score ranged from 0.28 to 0.96 (Figure 2). In total, 121 of 184 samples (65.8%) showed a probability score higher than 0.85, among which only the tumor origins of 6 samples were misclassified. The agreement rate was 95% (115/121). The remaining 63 specimens had a probability score less than 0.85, among which 27 samples were misclassified. The agreement rate is much lower (57.1%, 36/63).

The probability score for all of the samples have a correlation close to 0 (-0.003) with the ARG Ct value. A t-test showed no difference in the ARG Ct value between the correctly classified and misclassified group (*P* = 0.953). However, the probability score shows a significant difference between correct and incorrect groups (*P* = 1.794E-7).

### Performance Comparison of Primary and Metastasis Site

As previously described, except for 6 tumor types (skin, sarcoma, melanoma, mesothelioma, neuroendocrine, and lymphoma), 139 specimens were represented by 17 tumor types. Of those, 102 (73.4%) were the primary tumor, and 37 were metastases (26.6%). The classification accuracy for the primary site and metastasis site was 86.3% (n = 102) and 73.0% (n = 37), respectively.

For the metastasis group, we evaluated the biopsy site and prediction result. Two samples shared the same tumor type label. Both of the samples were gastro esophageal tumors metastasized to the ovary and were predicted as ovarian cancer. Additionally, tumor cells for both specimens were isolated by LCM. This misclassification could possibly be caused by technical mistakes in LCM. After removing these 2 samples, the agreement rate of the metastasis group was 77.1%.

In clinical practice, many unknown primary cancers were first identified in lymph nodes. In most of these cases, there were limited tumor cells and high contamination from lymphocytes. In our data set, 19 tumor specimens were obtained from lymph nodes. The overall accuracy was 68.4% (n = 19).

According to Fisher’s exact test, the performance for primary site, metastasis site and lymph node metastasis samples was not significantly different (86.3%, 73.0% and 68.4%; *P* = 0.060).

A one-way ANOVA test on average reference gene Ct values and probability scores showed no significant difference between primary site, metastasis and lymph node metastasis groups (ARG Ct *P* = 0.726; probability score *P* = 0.996).

### Performance Comparison of LCM and Scraping

In total, 127 of 184 specimens were dissected by LCM to enrich the tumor cells in the sections, and the remaining 57 specimens were scraped. The agreement rate is 91.2% (52/57) for the scraping group and 78.0% (99/127) for the LCM group, and this decrease in the LCM group is statistically significant (*P* = 0.03).

In addition, the ARG Ct value of the LCM group decreased significantly by 2.48 (t-test *P* = 3.75E-11) compared with the scraping group (mean difference). In general, the LCM samples have a 0.05 lower probability score than scrape samples, and this difference is also statistically significant (t-test *P* = 0.045).

### Tumor Grade and Assay Performance

Within the 184 samples, 90 have tumor grade information (48.9%, 90/184) in their pathological reports. In total, 12 were well differentiated (low grade or grade I), 28 were moderately differentiated (intermediate grade or grade II) and 50 were poorly differentiated or undifferentiated (high grade or grade III).

The agreement rate is 83.3% (10/12) in the grade I group, 75% (21/28) for grade II and 76% (38/50) for grade III (Table 4). However, the performance differences are not statistically significant (Fisher’s exact test *P* = 0.884). The ANOVA test also showed that both the probability score and the ARG Ct value were not significantly different among the three groups (*P* = 0.298 for ARG Ct; *P* = 0.096 for probability score).

**Table 4:** Performance of Grade III tumors.

	No. of cases	Correct predicted	Agreement rate
Germ-cell	1	1	1.00
Liver	1	1	1.00
Melanoma	1	1	1.00
Thyroid	1	1	1.00
Intestine	2	2	1.00
Breast	4	4	1.00
Ovary	7	6	0.86
Lung	6	5	0.83
Neuroendocrine	5	4	0.80
Urinary Bladder	5	4	0.80
Pancreas	3	2	0.67
Sarcoma	3	2	0.67
Gallbladder	2	1	0.50
Gastro esophageal	2	1	0.50
Head & Neck	4	2	0.50
Endometrium	3	1	0.33

### Performance Comparison with Previous Study

In a previous study by Ma et al., the THEROS CancerTYPE ID® assay (Version 1) was evaluated by testing 119 FFPE tumor samples. The overall accuracy was 82% within 32 tumor types from 26 tumor origins.

Compared to THERO CancerType ID® (Version 1), THERO CancerType ID® (Version 2) was developed later. The reference tumor database was expanded to 2,206 samples, and the associated algorithm was adjusted to enable the prediction of a modified list of 30 main tumor types and 54 histological subtypes. In the test set of 187 FFPE tumor samples representing 28 of the 30 main cancer types, the THERO CancerType ID® (Version 2) showed an overall sensitivity of 83% in previous validation in an American population [11].

In order to compare the performance of previous test set of Ma et al., with our data generated by THEROS CancerTYPE ID® assay (Version 2), previous tumor types of the CancerType ID (Version 1) were adjusted to obtain a comparable tumor type list. Some tumor subtypes from the same origin, such as lung small cell carcinoma, lung squamous, lung large-cell and adenocarcinoma, were combined together. While some other tumor types that had not been tested, such as brain tumor and meningioma, were removed. Finally, we obtained a list of 18 common main tumor types tested for both studies. Also the assay performance has been compared as illustrated in Table 5.

**Table 5:** A comparison on 18 common tumor types between Ma’s work with the data in this study.

	CancerType ID (Version 1)		FDUSCC-IM Lab (Version 2)	
	nr of sample tested	correctly predicted	nr of sample tested	correctly predicted
**Adrenal**	1	1	2	2
**Breast**	1	1	10	10
**Endometrium**	3	2	6	2
**Germ-cell**	9	7	10	10
**GIST**	3	3	6	6
**Intestine**	8	7	8	8
**Kidney**	4	4	8	7
**Liver**	2	2	6	6
**Lung**	11	5	8	7
**Lymphoma**	10	10	5	5
**Mesothelioma**	5	4	7	4
**Ovary**	5	5	14	9
**Pancreas**	3	3	7	5
**Prostate**	7	7	7	7
**Sarcoma**	13	10	13	10
**Skin**	11	9	6	5
**Thyroid**	3	3	10	10
**Urinary Bladder**	6	6	10	8
**Total**	105	89	143	121

In order to compare the overall performance of THERO CancerType ID® (Version 2) performed in American population with our study in Chinese population, 5 main tumor types that were not tested in this study were removed to obtain a comparable 23 tumor types in 171 samples.

The overall agreement rate is 84.8% (89/105) for the first test set of THERO CancerType ID® (Version 1) and 84.6% (121/143) in our study. A Pearson Chi-square test shows no significant difference (*P* = 0.975). (Table 5).

The comparison between the test set of 187 samples and this study showed an agreement rate of 83.6% (143/171) versus 82.1% (151/184). No significant difference was found (*P* = 0.697). (Table 6).

**Table 6:** A comparison on 23 tumor types between the test set of American population with the data in this study.

	CTID Version 2 (American population)		CTID Version 2 (Chinese population)	
	nr of sample tested	correctly predicted	nr of sample tested	correctly predicted
**Adrenal**	2	2	2	2
**Breast**	11	11	10	10
**Endometrium**	4	3	6	2
**Gallbladder**	6	4	5	4
**Gastro esophageal**	14	12	12	7
**Germ-cell**	6	6	10	10
**GIST**	1	1	6	6
**Head & Neck**	13	7	10	7
**Intestine**	16	10	8	8
**Kidney**	5	5	8	7
**Liver**	7	7	6	6
**Lung**	13	12	8	7
**Lymphoma**	10	10	5	5
**Melanoma**	5	4	5	4
**Mesothelioma**	2	2	7	4
**Neuroendocrine**	7	7	9	8
**Ovary**	6	5	14	9
**Pancreas**	8	5	7	5
**Prostate**	8	7	7	7
**Sarcoma**	6	6	13	10
**Skin**	9	6	6	5
**Thyroid**	5	5	10	10
**Urinary Bladder**	7	6	10	8
**Total**	171	143	184	151

Both study used the CancerType ID version 2.

## Discussion

To determine the origin of a tumor, clinicians should review the medical history, perform careful physical examination, perform different types of endoscopies and use several imaging devices, such as mammography, tomography, MRI or PET. IHC is also a current standard of care in tumor diagnosis. However, even with a growing panel of antibodies, the success rate of tumor origin determination is not completely satisfactory. A meta-analysis showed that an extensive IHC workup correctly identified the primary site for 66% of all metastatic specimens [3]. Gene expression profiling has been used for tumor classification in many studies [6]–[9], [13]. However, in clinical practice, doctors will encounter all types of tumor samples, from lymph node metastasis to primary cancer, from fine needle biopsy to surgery resection, from well-differentiated to poorly differentiated tumors and with a variety of RNA quantity and integrity. A molecular profiling assay that can be applied to all types of samples is required. Moreover, due to the complexity and quantitative nature of such technology, a standard sample inclusion step and sample treatment process should be applied to obtain a comparable and reproducible performance result.

In this study, we have evaluated the performance of the THEROS CancerTYPE ID®, a real-time PCR-based 92-gene mRNA expression profiling panel, with 184 FFPE tissue specimens from Chinese patients. This study is performed blindly and independently in an outside lab, bioTheranostics Inc., using stored specimens in the tissue bank of FDUSCC. This study demonstrated that the CTID assay could be carefully performed outside of the CLIA lab in the United States with different samples taken from Chinese population and show comparable performance. In addition, we also tried to explore the effect of several sample subgroups.

Published gene expression-based CUP assays often have some sample inclusion criteria on tumor representation, minimum necrosis and total RNA quality. For example, in the work of Rosenfeld et al., most samples included (>90%) had at least 50% tumor in the section area, and 5 µg of total RNA was needed [9]. In the work of Monzon et al., a visual examination by pathologists was required, and at least 60% tumor representation and <20% necrosis were included [7], [8]. In the previous evaluation study of the THEROS CancerTYPE ID® by Ma et al., the average tumor content of all samples was approximately 65% [6].

With the application of LCM, THEROS CancerTYPE ID® (Version 2) is able to treat specimens with disseminated tumor cells, such as lymph node metastasis. In our data, although a performance decrease is observed in the LCM group, it still showed a 78% agreement rate.

However, there are technical difficulties when using LCM. Due to the highly scattered tumor cells and low tumor representation in some specimens, the low quantity of RNA, contamination with surrounding cells, such as fibrocytes, lymphocytes or necrotic areas, and RNA degradation during staining and dissection, qRT-PCR results can be changed.

In our data, the low integrity and quantity of RNA did not significantly affect the classification results. We observed a decrease in the sensitivity with an increase in ARG Ct value, but this difference was not statistically significant. In addition, no differences in ARG Ct were found between the correctly classified and misclassified group.

Contamination was also found in at least 2 samples. Both of these samples are gastro esophageal tumor metastases to the ovary. The specimens were taken from the metastatic site, and both were predicted as ovarian cancer. Considering these technical difficulties, a decrease in the classification performance from 91% in scraped samples to 78% in LCM specimens seems unavoidable.

In clinical practice, metastases often increase the demand for the primary tumor origin to be identified quickly and accurately. However, many previous studies had already described the change in morphology, IHC and mRNA profile. This change will result in a decrease in classification accuracy by pathologists or molecular tests based on gene expression profiles. In our data, primary tumors showed the highest sensitivity, with an agreement rate of 86.3%. All of the 37 metastatic tumors showed a 73% agreement rate. For some patients, the only metastasis site identified is the lymph node. We also calculated the agreement rate of the 19 lymph node metastasis specimens and obtained 68.4% sensitivity. However, this decrease is not significant (*P* = 0.06).

The metastasis group had more LCM specimens. It is hard to determine whether the performance decrease is mainly caused by metastasis-related expression profile changes or contamination during LCM. In addition, the limited sample size within the metastasis group made the result less convincing.

Tumor grade may also change the morphology and expression profile. Poorly differentiated tumors are sometimes difficult to identify and will cause misdiagnosis. However, in our data, the 50 grade III tumor samples had a 76% agreement rate, and the performance differences between the three grades were not statistically significant (P = 0.884).

Previously, two validations had been performed by using samples for which the tumor origin was already identified in American population. Our study showed the overall performance of THEROS CancerTYPE ID® (Version 2) in Chinese population are comparable to the previous test by THEROS CancerTYPE ID® (Version 1) [6] and the resent test by THEROS CancerTYPE ID® (Version 2) in American population [11]. However, the performance for some types of cancer, such as endometrial and ovarian cancer, was decreased in the Chinese population. Tumors such as lung cancer and intestine cancer showed an increase in performance in these validation tests. However, due to the limited samples of each tumor type and histology type, it is difficult to confirm whether this performance difference was caused by an expression profile difference between different populations or the histology type tested.

Although a total of 184 samples were included in the study, for certain types of tumor with small samples, the analysis have been unable to achieve sufficient statistical power. Such as the adrenal cancer, only 2 samples were tested. Additional study with enlarged sample size for certain types of tumor in deed necessary in the future.

### Conclusion

We evaluated the performance of the THEROS CancerTYPE ID® in 184 Chinese tumor specimens of 23 types. The assay was performed blindly and independently. A low sample inclusion criterion was set (at least 300 tumor cells), and no sample was excluded during the inclusion and experimental stage.

A comparable result with previous work was generated, with a total performance of 82.1%. Specifically, the specimens with a high probability score (>0.85) had a high chance (agreement rate  = 95%) of being correctly predicted. Moreover, no statistically significant performance difference was observed between primary and metastatic specimens, and no difference was observed among three tumor grades. The use of LCM makes the THEROS CancerTYPE ID® accessible to almost all of the cancer patients with different statuses of tumor specimens. These characteristics make the THEROS CancerTYPE ID® a valuable tool in clinical practice.
